# Outcomes in Antiplatelet‐Associated Intracerebral Hemorrhage in the TICH‐2 Randomized Controlled Trial

**DOI:** 10.1161/JAHA.120.019130

**Published:** 2021-02-15

**Authors:** Zhe Kang Law, Michael Desborough, Ian Roberts, Rustam Al‐Shahi Salman, Timothy J. England, David J. Werring, Thompson Robinson, Kailash Krishnan, Robert Dineen, Ann Charlotte Laska, Nils Peters, Juan Jose Egea‐Guerrero, Michal Karlinski, Hanne Christensen, Christine Roffe, Daniel Bereczki, Serefnur Ozturk, Jegan Thanabalan, Rónán Collins, Maia Beridze, Philip M. Bath, Nikola Sprigg

**Affiliations:** ^1^ Stroke Trials Unit Division of Clinical Neuroscience University of Nottingham United Kingdom; ^2^ Department of Medicine National University of Malaysia Kuala Lumpur Malaysia; ^3^ Haemophilia and Thrombosis Centre Guy’s and St Thomas’ NHS Foundation Trust London United Kingdom; ^4^ Clinical Trials Unit London School of Hygiene & Tropical Medicine London United Kingdom; ^5^ Centre for Clinical Brain Sciences University of Edinburgh United Kingdom; ^6^ Vascular Medicine Division of Medical Sciences & GEM Royal Derby Hospital Centre University of Nottingham United Kingdom; ^7^ Stroke Research Centre UCL Queen Square Institute of Neurology London United Kingdom; ^8^ Department of Cardiovascular Sciences and National Institute for Health Research Biomedical Research Centre University of Leicester United Kingdom; ^9^ Nottingham University Hospitals NHS Trust Nottingham United Kingdom; ^10^ Radiological Sciences University of Nottingham United Kingdom; ^11^ National Institute for Health Research Nottingham Biomedical Research Centre Nottingham United Kingdom; ^12^ Department of Clinical Sciences Karolinska Institutet Danderyd Hospital Sweden; ^13^ Neurology and Stroke Center Klinik Hirslanden Zürich Switzerland; ^14^ Neurology and Neurorehabilitation Unit University Center for Medicine of Aging Felix Platter‐Hospital Basel Switzerland; ^15^ Department of Neurology and Stroke Center University Hospital Basel and University of Basel Switzerland; ^16^ NeuroCritical Care Unit Virgen del Rocio University Hospital Seville Spain; ^17^ Institute of Psychiatry and Neurology Warsaw Poland; ^18^ Department of Neurology Bispebjerg Hospital and University of Copenhagen Denmark; ^19^ Stroke Research Faculty of Medicine and Health Sciences Keele University Stoke‐on‐Trent United Kingdom; ^20^ Department of Neurology Semmelweis University Budapest Hungary; ^21^ Department of Neurology Selcuk University Faculty of Medicine Konya Turkey; ^22^ Division of Neurosurgery Department of Surgery National University of Malaysia Kuala Lumpur Malaysia; ^23^ Tallaght University Hospital Dublin Republic of Ireland; ^24^ The First University Clinic of Tbilisi State Medical University Tbilisi Georgia

**Keywords:** cerebral hemorrhage, antiplatelet, tranexamic acid, hematoma expansion, randomized controlled trial, Cerebrovascular Disease/Stroke, Intracranial Hemorrhage, Clinical Studies, Platelets, Treatment

## Abstract

**Background:**

Antiplatelet therapy increases the risk of hematoma expansion in intracerebral hemorrhage (ICH) while the effect on functional outcome is uncertain.

**Methods and Results:**

This is an exploratory analysis of the TICH‐2 (Tranexamic Acid in Intracerebral Hemorrhage‐2) double‐blind, randomized, placebo‐controlled trial, which studied the efficacy of tranexamic acid in patients with spontaneous ICH within 8 hours of onset. Multivariable logistic regression and ordinal regression were performed to explore the relationship between pre‐ICH antiplatelet therapy, and 24‐hour hematoma expansion and day 90 modified Rankin Scale score, as well as the effect of tranexamic acid. Of 2325 patients, 611 (26.3%) had pre‐ICH antiplatelet therapy. They were older (mean age, 75.7 versus 66.5 years), more likely to have ischemic heart disease (25.4% versus 2.7%), ischemic stroke (36.2% versus 6.3%), intraventricular hemorrhage (40.2% versus 27.5%), and larger baseline hematoma volume (mean, 28.1 versus 22.6 mL) than the no‐antiplatelet group. Pre‐ICH antiplatelet therapy was associated with a significantly increased risk of hematoma expansion (adjusted odds ratio [OR], 1.28; 95% CI, 1.01–1.63), a shift toward unfavorable outcome in modified Rankin Scale (adjusted common OR, 1.58; 95% CI, 1.32–1.91) and a higher risk of death at day 90 (adjusted OR, 1.63; 95% CI, 1.25–2.11). Tranexamic acid reduced the risk of hematoma expansion in the overall patients with ICH (adjusted OR, 0.76; 95% CI, 0.62–0.93) and antiplatelet subgroup (adjusted OR, 0.61; 95% CI, 0.41–0.91) with no significant interaction between pre‐ICH antiplatelet therapy and tranexamic acid (P interaction=0.248).

**Conclusions:**

Antiplatelet therapy is independently associated with hematoma expansion and unfavorable functional outcome. Tranexamic acid reduced hematoma expansion regardless of prior antiplatelet therapy use.

**Registration:**

URL: https://www.isrctn.com; Unique identifier: ISRCTN93732214.

**Nonstandard Abbreviations and Acronyms**
DASH: Desmopressin for Reversal of Antiplatelet Drugs in Stroke Due to Hemorrhage
ICH: intracerebral hemorrhage
TICH‐2: Tranexamic Acid in Intracerebral Hemorrhage‐2

**Clinical Perspective**

**What Is New?**

- Prior antiplatelet therapy is associated with an increased risk of hematoma expansion, neurological deterioration, death, and unfavorable functional outcome in spontaneous intracerebral hemorrhage.
- Tranexamic acid reduced hematoma expansion but did not improve day 90 functional outcome in patients with intracerebral hemorrhage regardless of prior antiplatelet therapy.

**What Are the Clinical Implications?**

- The use of tranexamic acid in patients with antiplatelet‐associated intracerebral hemorrhage needs to be further studied.

Approximately 25% of patients with intracerebral hemorrhage (ICH) are on antiplatelet treatment before ICH.^1^ Antiplatelet therapy impairs hemostasis, which may lead to hematoma expansion.^2^ In addition, patients taking antiplatelet therapy were more likely to have a previous ischemic stroke, ischemic heart disease, or peripheral vascular disease leading to more post‐ICH complications. Nevertheless, the effect of antiplatelet therapy on clinical outcomes is uncertain. Some studies reported that pre‐ICH antiplatelet therapy increased the risk of neurological deterioration, death, and poor functional outcome,^1^, ^3^, ^4^, ^5^ while others did not.^1^, ^6^, ^7^, ^8^, ^9^, ^10^ Antiplatelet therapy was reported to be associated with larger baseline hematoma volume,^6^ intraventricular hemorrhage,^11^ and hematoma expansion^2^, ^12^ in some studies, though others have shown that antiplatelet therapy did not have significant effects on hematoma characteristics.^9^, ^10^ A large individual patient data meta‐analysis (5435 patients, 36 observational cohorts) concluded that antiplatelet therapy, as well as shorter time from onset to imaging, larger baseline hematoma volume, and pre‐ICH anticoagulant therapy, independently increased the risk of hematoma expansion.^2^

There is currently no specific hemostatic therapy for antiplatelet‐associated intracerebral hemorrhage. Platelet transfusion was harmful in 1 randomized controlled trial.^13^ The effects of tranexamic acid on antiplatelet‐associated ICH have not been studied in a randomized controlled setting.

In the primary publication of the TICH‐2 (Tranexamic Acid in Intracerebral Hemorrhage‐2) trial, there was no significant difference in the primary outcome of ordinal shift in day 90 modified Rankin scale between tranexamic acid and placebo arm, despite a reduction in hematoma expansion.^14^ In the current analysis, we aimed to explore the relationship between pre‐ICH antiplatelet therapy and hematoma characteristics and functional outcome in spontaneous ICH; and to explore the effect of tranexamic acid in patients taking antiplatelet therapy. We hypothesized that antiplatelet therapy increases the risk of hematoma expansion and poor functional outcome and that tranexamic acid has a beneficial effect on hematoma expansion and outcome in patients taking antiplatelet therapy.

## Methods

### Data Sharing

The trial data can be shared upon reasonable request to the corresponding author and trial steering committee.

### Study Design and Population

We analyzed data from the TICH‐2 trial, which was a prospective multicenter randomized double‐blind placebo‐controlled trial. Patients aged >18 years with spontaneous ICH presenting within 8 hours of symptoms onset were eligible, while secondary (macrovascular or structural) causes of ICH were excluded. Patients who were taking anticoagulants at the time of ICH were excluded as well. Recruited patients were randomized 1:1 with minimization on key prognostic factors and stratification by country to receive 2 g of intravenous tranexamic acid or matching placebo. Detailed descriptions of the trial were previously published.^14^, ^15^, ^16^

### Outcomes

We compared hematoma expansion at 24‐hour, early outcomes (death and neurological deterioration within 7 days), and day 90 outcomes (modified Rankin Scale, disability, cognition, depression, and quality‐of‐life scores) between the antiplatelet and no‐antiplatelet groups. In the antiplatelet subgroup analysis, we explored the effects of tranexamic acid on hematoma characteristics and clinical outcomes. These outcomes were prespecified in the trial protocol.^15^

### Definitions

Hematoma expansion was defined as an increase of >6 mL or >33% in hematoma volume at 24‐hour computed tomography scan compared with baseline hematoma volume, measured using semiautomated segmentation technique with ITK‐SNAP version 3.6.0 software.^17^, ^18^ Details on image acquisition and analyses were previously described.^19^ A Modified Rankin Scale score of 4 to 6 was considered poor functional outcome.

### Statistical Analysis

Descriptive analysis was performed using the Student *t*, Mann‐Whitney *U*, and chi‐squared tests. Multiple linear regression, multivariable binary logistic regression, and ordinal logistic regression analyses with adjustment of covariates (which are key prognostic factors: age, sex, systolic blood pressure, National Institutes of Health Stroke Scale, onset to randomization time, intraventricular hemorrhage, and baseline hematoma volume, as well as country of recruitment) were performed to determine the effect of antiplatelet therapy on outcomes and also the effect of tranexamic acid on outcomes in the antiplatelet subgroup. The selection of covariates was prespecified in our statistical analysis plan^18^ and were similar to the selection of covariates as reported in the main result paper.^14^ We report the odds ratio for effect of tranexamic acid for the antiplatelet and no‐antiplatelet subgroups separately and also the *P* value for interaction between antiplatelet intake status and tranexamic acid treatment. Statistical analyses were performed using SPSS version 24 (IBM, Armonk, NY). A *P* value of <0.05 was considered statistically significant.

### Ethics Approval

This study was approved by the national and institutional ethics review committees of participating countries/centers. Written informed consent was obtained from the patient(s) or their representatives.

## Results

Of the 2325 patients recruited, 611 (26.3%) were taking antiplatelet therapy at the time of ICH, while 1713 (73.7%) were not; information on antiplatelet intake was not available in 1 patient. Data on functional outcome and hematoma expansion were available in 2306 and 2077 patients, respectively (Figure S1). Patients who were taking antiplatelet therapy were significantly older, more likely to have previous stroke or ischemic heart disease, and had more severe stroke and lower systolic blood pressure on admission (Table 1). Patients with pre‐ICH antiplatelet therapy had significantly larger baseline hematoma volume and were more likely to have intraventricular hemorrhage, subarachnoid extension, old infarct(s), leukoaraiosis, and cerebral atrophy on baseline computed tomography scan. There was no difference in the proportion of patients with supratentorial lobar, supratentorial deep, and infratentorial hematoma between the antiplatelet and no‐antiplatelet groups. Within the antiplatelet group, characteristics of patients who received tranexamic acid and placebo were similar (Table 1).

**Table 1:** Baseline Characteristics of Patients With Intracerebral Hemorrhage Associated With Versus Without Prior Antiplatelet Therapy

Characteristics	Antiplatelet (n=611)	No Antiplatelet (n=1713)	*P* Value	Antiplatelet Subgroup		*P* Value
				Tranexamic Acid (316, 51.7%)	Placebo (295, 48.3%)	
Clinical						
Age, mean (SD), y	75.7 (11.3)	66.5 (13.8)	0.001	75.8 (11.3)	75.5 (11.3)	0.725
Sex, male	366 (59.9%)	934 (54.5%)	0.071	190 (60.1%)	176 (59.7%)	0.907
Onset to CT time, h, mean (SD)	2.4 (1.3)	2.2 (1.3)	0.002	2.5 (1.4)	2.4 (1.3)	0.407
Onset to randomization time, h, mean (SD)	4.1 (1.7)	3.9 (1.7)	0.022	4.1 (1.8)	4.0 (1.7)	0.563
Premorbid mRS, median (IQR)	0 (0–2)	0 (0–0)	<0.001	0 (0–2)	0 (0–2)	0.480
Glasgow Coma scale, median (IQR)	14 (12–15)	15 (12–15)	0.002	14 (12–15)	14 (12–15)	0.592
NIHSS, median (IQR)	13 (7–20)	12 (7–18)	0.018	14 (6–20)	13 (8–20)	0.816
Admission SBP, mean (SD), mm Hg	171.3 (27.4)	176.0 (30.5)	<0.001	171.2 (28.3)	171.5 (26.4)	0.912
24‐h SBP, mean (SD), mm Hg*	149.1 (20.8)	149.4 (20.1)	0.743	147.8 (21.2)	150.4 (20.3)	0.144
Previous stroke or TIA	221 (36.2%)	108 (6.3%)	0.001	110 (35.4%)	111 (38.1%)	0.480
Previous intracerebral hemorrhage	27 (4.5%)	99 (5.8%)	0.210	14 (4.5%)	13 (4.4%)	0.983
Previous ischemic heart disease	155 (25.4%)	47 (2.7%)	0.001	84 (27.2%)	71 (24.3%)	0.422
Treatment with tranexamic acid	316 (51.7%)	844 (49.3%)	0.299	…	…	
Country (United Kingdom)	518 (84.8%)	1391 (81.2%)	0.048	266 (84.2%)	252 (85.4%)	0.668
On antiplatelet therapy, day 7	10 (1.7%)	2 (0.1%)	<0.001	2 (0.6%)	8 (2.7%)	0.046
On antiplatelet therapy, day 90	31 (9.6%)	29 (2.4%)	<0.001	20 (12.4%)	11 (6.8%)	0.089
Radiological (CT scan)						
Location						
Supratentorial lobar	189 (31.7%)	499 (29.6%)	0.329	98 (31.7%)	91 (31.7%)	0.998
Supratentorial deep	369 (61.9%)	1082 (64.1%)	0.332	176 (61.3%)	193 (62.5%)	0.775
Infratentorial	38 (6.4%)	106 (6.3%)	0.936	20 (7.0%)	18 (5.8%)	0.568
Baseline hematoma volume, mean (SD), mL	28.1 (31.5)	22.6 (25.3)	<0.001	29.9 (33.7)	26.1 (28.9)	0.134
Intraventricular hemorrhage	241 (40.2%)	466 (27.5%)	<0.001	131 (42.3)	110 (38.1)	0.295
Subarachnoid hemorrhage	95 (15.5%)	204 (11.9%)	0.023	52 (16.8)	43 (14.9)	0.515
Old infarct(s)	438 (72.8%)	955 (56.4%)	<0.001	223 (71.5)	215 (74.1)	0.463
Leukoaraiosis	350 (58.1%)	707 (41.7%)	<0.001	184 (59.0)	166 (57.2)	0.667
Cerebral atrophy	584 (97.0%)	1515 (89.4%)	<0.001	304 (97.4)	280 (96.6)	0.524

Analyses by chi‐square, Student *t* test, or Mann‐Whitney *U* test. CT indicates computed tomography; IQR, interquartile range; mRS, modified Rankin Scale; NIHSS, National Institutes of Health Stroke Scale; SBP, systolic blood pressure; and TIA, transient ischemic attack.

* No data available on SBP between admission and 24 hours.

**Figure 1:**
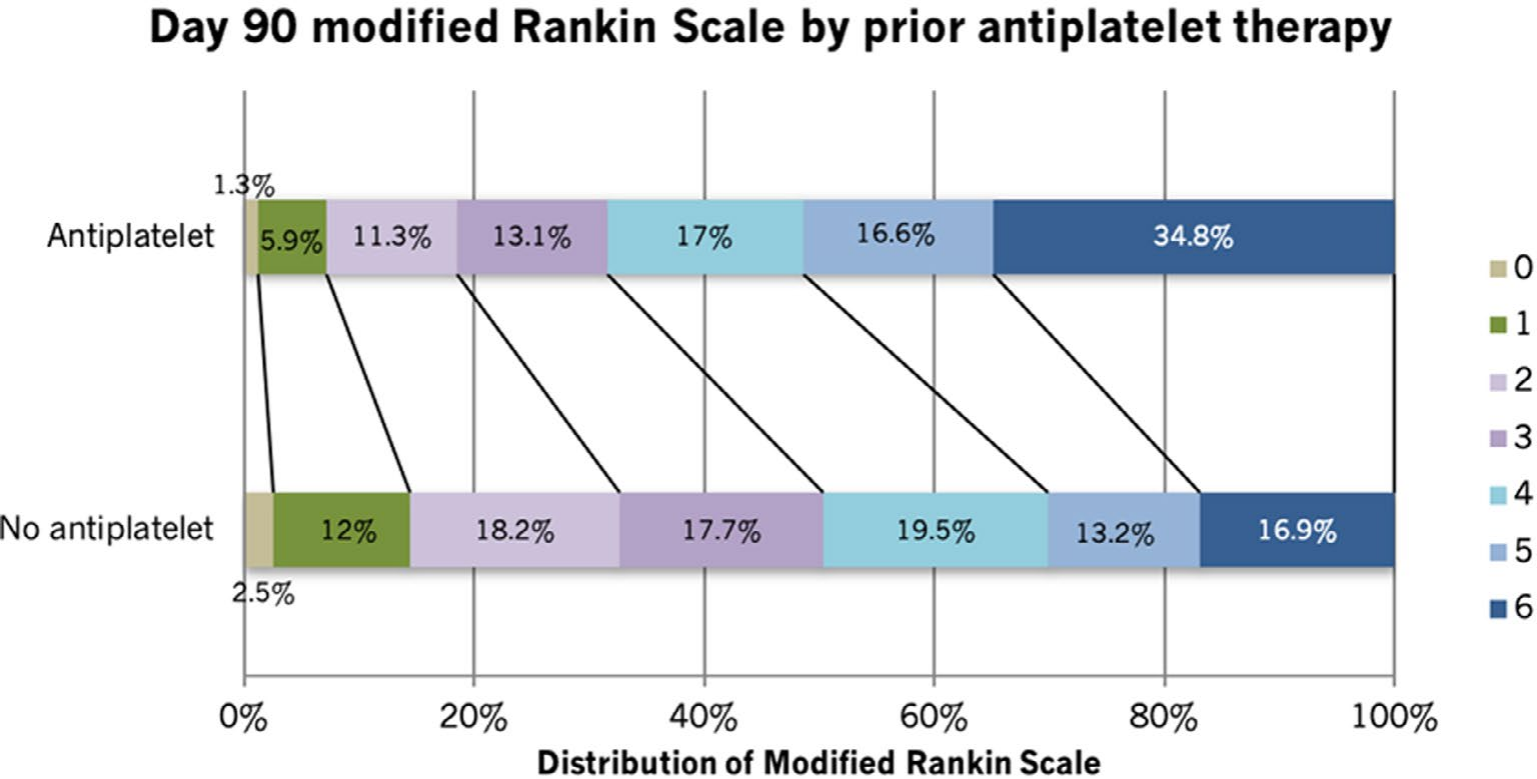
Functional outcome at day 90 after intracerebral hemorrhage in antiplatelet vs no antiplatelet subgroups (adjusted common odds ratio, 1.58; 95% CI 1.32–1.91; *P*<0.001). Analysis was ordinal logistic regression, adjusted for age, sex, systolic blood pressure, National Institutes of Health Stroke Scale, onset to randomization time, intraventricular hemorrhage, treatment group, and country.

At 24 hours, patients with pre‐ICH antiplatelet therapy had significantly larger hematoma volume, greater absolute hematoma growth, and hematoma expansion compared with the no‐antiplatelet group (Table 2). The risks of intraventricular and subarachnoid extension of hemorrhage were significantly increased as well. The risks of neurological deterioration and death in the first 7 days were significantly higher in the antiplatelet group even after adjusting for key prognostic factors, as stated in the Methods section. At day 90, the risks of death (adjusted odds ratio [aOR], 1.63; 95% CI, 1.25–2.11; *P*<0.001) and poor functional outcome (aOR, 1.52; 95% CI, 1.17–1.98; *P*=0.002) were significantly higher in the antiplatelet group compared with the no‐antiplatelet group. Similarly, disability, depression, quality of life, and cognition scores were significantly worse in the antiplatelet group (Table 2). The proportion of patients with new stroke, acute coronary syndrome, and venous thromboembolism did not differ between the 2 groups. Pre‐ICH antiplatelet therapy was associated with a significant shift toward unfavorable outcome in ordinal regression analysis (aOR, 1.58; 95% CI, 1.32–1.91; *P*<0.001; Figure 1).

**Table 2:** Outcomes After Intracerebral Hemorrhage Associated With Versus Without Prior Antiplatelet Therapy

Outcomes	Antiplatelet	No Antiplatelet	OR/MD (95% CI)*	*P* Value
Radiological, 24‐h CT				
Hematoma volume, mean (SD), mL	33.8 (39.3)	24.8 (27.4)	9.0 (5.4, 12.6)	<0.001
Hematoma growth, mean (SD), mL	7.3 (21.4)	3.3 (13.5)	3.2 (1.7, 4.8)	<0.001
Hematoma expansion^†^	165 (30.7%)	403 (25.1%)	1.28 (1.01, 1.63)	0.046
New interventricular hemorrhage	53 (9.8%)	126 (7.8%)	1.58 (1.12, 2.24)	0.010
Subarachnoid extension^‡^	47 (8.7%)	75 (4.7%)	1.99 (1.36, 2.91)	<0.001
Day 7				
Neurological deterioration^§^	245 (40.1%)	402 (23.5%)	1.52 (1.21, 1.91)	<0.001
Death	106 (17.3%)	118 (6.9%)	1.94 (1.40, 2.69)	<0.001
DNAR	215 (35.2%)	294 (17.2%)	1.64 (1.25, 2.15)	<0.001
Day 90				
Death	212 (34.8%)	287 (16.9%)	1.63 (1.25, 2.11)	<0.001
mRS >3	417 (68.4%)	841 (49.6%)	1.52 (1.17, 1.98)	0.002
Barthel Index, mean (SD)	37.5 (44.2)	58.7 (42.4)	−6.5 (−9.7, −3.4)	<0.001
EQ5D, mean (SD)	0.23 (0.37)	0.38 (0.40)	−0.05 (−0.08, −0.02)	0.003
TICS‐M, mean (SD)	7.5 (11.7)	16.0 (12.2)	−2.2 (−3.3, −1.0)	<0.001
ZDS, mean (SD)	81.7 (28.1)	62.3 (28.5)	6.3 (3.5, 9.1)	<0.001
Safety events^‖^				
New stroke	4 (0.7%)	11 (0.6%)	…	0.544
New acute coronary syndrome	1 (0.2%)	4 (0.2%)	…	1.000
New venous thromboembolism	6 (1.6%)	25 (1.8%)	…	1.000

DNAR indicates do not attempt resuscitation; EQ5D, Euro Quality of Life; MD, mean difference; mRS, modified Rankin Scale; NIHSS, National Institutes of Health Stroke Scale; OR, odds ratio; TICS‐M, modified telephone interview cognitive status; and ZDS, Zung depression scale.

* Analyses are linear or binary logistic regression adjusted for age, sex, systolic blood pressure, NIHSS, onset to randomization time, intraventricular hemorrhage, treatment group and country, except for ^†^hematoma expansion and ^‡^subarachnoid extension where baseline hematoma volume and presence of baseline subarachnoid hemorrhage were additional covariates respectively. The selection of variables was based on the main analyses, where minimization factor and stratification by country were prespecified covariates.

^§^ Neurological deterioration: increase of NIHSS ≥4 or decrease in Glasgow Coma Scale score of ≥2.

^‖^ Fisher's exact test.

We explored the effect of tranexamic acid on outcomes in the antiplatelet group. Tranexamic acid significantly reduced hematoma expansion (aOR, 0.61; 95% CI, 0.41–0.91; *P*=0.015) but did not have significant effects on all clinical outcomes (Table 3). Tranexamic acid did not have a significant benefit on shift of modified Rankin Scale at day 90 (aOR, 0.92; 95% CI, 0.68–1.25; *P*=0.589; ordinal regression analysis with adjustment for age, sex, systolic blood pressure, National Institutes of Health Stroke Scale, onset to randomization time, intraventricular hemorrhage, and country; Figure S2).

**Table 3:** Effect of Tranexamic Acid on Outcomes for Participants With Intracerebral Hemorrhage Associated With Prior Antiplatelet Therapy

Outcomes	Tranexamic Acid (n=316)	Placebo (n=295)	OR/MD (95% CI)*	*P* Value
Baseline CT				
Hematoma volume, mL, mean (SD)	29.9 (33.7)	26.1 (28.9)	3.8 (−1.2 to 8.9)	0.136
24‐h CT				
Hematoma volume, mL, mean (SD)	35.0 (40.1)	32.4 (38.4)	2.6 (−4.1 to 9.3)	0.442
Hematoma growth, mL, mean (SD)	6.0 (18.9)	8.7 (23.8)	−3.0 (−6.5 to 0.5)	0.088
Hematoma expansion^†^	76 (27.2%)	89 (34.5%)	0.61 (0.41 to 0.91)	0.015
Day 7, n (%)				
Neurological deterioration	127 (40.4)	139 (47.1)	0.71 (0.49 to 1.03)	0.068
Death	53 (16.8)	53 (18.0)	0.81 (0.51 to 1.30)	0.388
DNAR	111 (35.1)	104 (35.3)	0.93 (0.62 to 1.40)	0.726
Day 90				
Death, n (%)	110 (34.8)	102 (34.7)	0.89 (0.60 to 1.33)	0.570
mRS >3, n (%)	215 (68.0)	202 (68.7)	0.94 (0.60 to 1.47)	0.782
Barthel index, mean (SD)	37.1 (43.9)	37.9 (44.5)	0.6 (−4.6 to 5.7)	0.828
EQ5D, mean (SD)	0.23 (0.37)	0.23 (0.37)	0.01 (−0.04 to −0.06)	0.758
TICS‐M, mean (SD)	7.0 (11.3)	8.0 (12.1)	−0.5 (−2.3 to 1.2)	0.550
ZDS, mean (SD)	81.6 (28.4)	81.8 (27.9)	−0.6 (−5.1 to 3.9)	0.805
Safety events,^‡^ n (%)				
New stroke	2 (1.0)	2 (1.1)	…	1.000
New acute coronary syndrome	0 (0)	1 (0.6)	…	0.487
New venous thromboembolism	4 (2.1)	2 (1.1)	…	0.686

DNAR indicates do not attempt resuscitation; EQ5D, Euro Quality of Life; NIHSS, National Institutes of Health Stroke Scale; MD, mean difference; mRS, modified Rankin Scale; OR, odds ratio; TICS‐M, modified telephone interview cognitive status; and ZDS, Zung depression scale.

* Binary logistic regression or multiple linear regression adjusted for age, sex, systolic blood pressure, National Institute of Health Stroke Scale, onset to randomization time, intraventricular hemorrhage, and country with addition of baseline hematoma volume for hematoma expansion.

^†^ Hematoma expansion: >6 mL or >33% increase in volume from baseline.

^‡^ Fisher's exact test.

**Figure 2:**
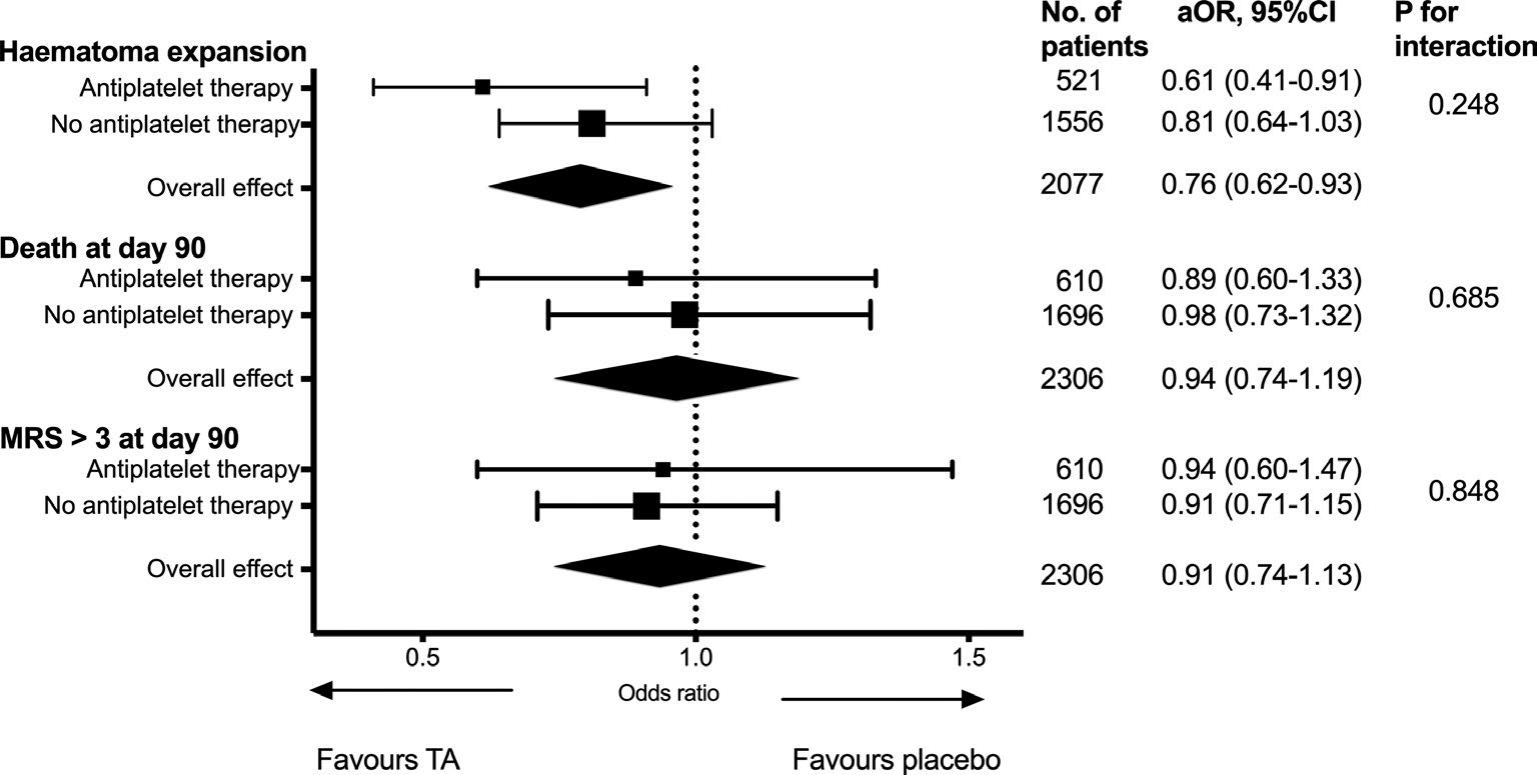
Effect of tranexamic acid on hematoma expansion, death and death or dependence (modified Rankin Scale score [mRS] >3) at day 90 stratified by prior use of antiplatelet therapy. Analysis was multivariable binary logistic regression with adjustment for age, sex, systolic blood pressure, National Institutes of Health Stroke Scale, onset to randomization time, intraventricular hemorrhage, and country for outcome of death and mRS >3 at day 90 with additional variable of baseline hematoma volume for hematoma expansion. Figure differed from previously published^14^ as current analysis included additional clinical scans; and the presence of intraventricular hemorrhage was based on expert radiologists' adjudication rather than investigator reported. aOR indicates adjusted odds ratio; and mRS, modified Rankin Scale.

We examined the effect of tranexamic acid on hematoma expansion, death, and modified Rankin Scale >3 at day 90 stratified by pre‐ICH antiplatelet versus no antiplatelet. Overall, tranexamic acid significantly reduced the risk of hematoma expansion but not death and dependency. Tranexamic acid significantly reduced hematoma expansion in the antiplatelet subgroup (aOR, 0.61; 95% CI, 0.41–0.91) but not in the no‐antiplatelet subgroup (aOR, 0.81; 95% CI, 0.64–1.03), though there was no significant statistical interaction (*P*=0.248; Figure 2).

## Discussion

In this exploratory analysis of the TICH‐2 trial, pre‐ICH antiplatelet therapy was associated with an increased risk of hematoma expansion, as well as intraventricular and subarachnoid extension of hemorrhage. This suggests that antiplatelet therapy is associated with significant impairment of hemostasis in ICH. However, the increased risk of bleeding may be attributed to other confounders such as older age and leukoaraiosis, which were more common in patients taking antiplatelet therapy.

Our findings are in agreement with a large individual patient data meta‐analysis that included 5435 patients from 36 cohorts, which concluded pre‐ICH antiplatelet therapy as an independent predictor of hematoma expansion.^2^ On the other hand, several large studies found that pre‐ICH antiplatelet therapy did not result in hematoma expansion.^9^, ^10^ This may be because of the heterogenous population that antiplatelet‐associated ICH represents or that the definition of hematoma expansion in terms of changes in volume or timing of follow‐up neuroimaging was not standardized. For example, one study that did not find antiplatelet therapy as significant risk factor for hematoma expansion used a cutoff of ≥12.5 mL as compared with >6 mL in our study.^9^

Pre‐ICH antiplatelet therapy was associated with worse early outcome of neurological deterioration and death within 7 days, as well as death and functional outcome at 90 days. Several previous studies concluded similarly,^1^, ^3^, ^4^, ^5^ while others disputed the effect of antiplatelet therapy on longer‐term outcome.^1^, ^6^, ^7^, ^8^, ^9^, ^10^ Worse functional outcome and death in patients taking antiplatelet therapy may be confounded by adverse prognostic factors such as older age and higher prevalence of vascular disease.^20^ In this analysis, we included key prognostic factors as covariates in the regression analyses, accounting for possible baseline confounders such as age and National Institutes of Health Stroke Scale. In addition, pre‐ICH antiplatelet therapy was associated with worse day 90 Barthel Index, cognitive, depression, and quality‐of‐life scores. The negative effect of antiplatelet therapy in ICH is likely related to a larger baseline hematoma and increased risk of hematoma expansion in the antiplatelet group. These findings highlight the need to prioritize research and clinical care in antiplatelet‐associated ICH. An example of clinical trials that specifically targets antiplatelet‐associated ICH is the DASH (Desmopressin for Reversal of Antiplatelet Drugs in Stroke Due to Hemorrhage) trial that involved the use of desmopressin in these patients (NCT03696121).^21^

Another important finding is that there was no heterogeneity of hemostatic effect of tranexamic acid across the antiplatelet and no‐antiplatelet subgroups. In fact, the reduction in aOR for hematoma expansion was numerically greater in the antiplatelet subgroup (aOR, 0.61; 95% CI, 0.41–0.91) compared with the no‐antiplatelet subgroup (aOR, 0.81, 95% CI 0.64–1.03). The absolute 24‐hour hematoma volume reduction with tranexamic acid was greater as well in the antiplatelet subgroup (3.0 mL, Table 3) compared with the reduction in all patients (1.4 mL) as reported in the main publication.^14^ However, as the *P* for interaction was not significant (*P*=0.248), these findings do not support a greater benefit of tranexamic acid in the antiplatelet subgroup compared with the no‐antiplatelet subgroup.

We hypothesized that there are several mechanisms of how tranexamic acid, an antifibrinolytic agent, may have exerted hemostatic effect in antiplatelet‐associated ICH despite not having a direct platelet‐enhancing effect. Antiplatelet therapy such as aspirin acts predominantly by inhibiting platelet aggregation, but it may also inhibit acetylate lysine residues in fibrinogen leading to increased fibrin clot permeability and increased vulnerability to fibrinolysis,^22^, ^23^ which may have an important role in hematoma expansion in ICH.^24^ Tranexamic acid acts by preventing the breakdown of stable fibrin clots. Therefore, it is possible that antiplatelet‐treated patients were more vulnerable to fibrinolysis due to disturbance in fibrin formation and hence may benefit from tranexamic acid. Other mechanisms for the effect of tranexamic acid have been proposed such as inhibiting plasmin‐induced platelet activation but there are conflicting results between studies.^25^, ^26^, ^27^ In view of these, the effect of tranexamic acid on hematoma expansion in the antiplatelet group should be further explored in future studies.

One advantage of the current study is a relatively large prospective cohort compared with previous studies and an excellent availability of data on functional outcome (99%) and hematoma expansion (≈90%). In comparison, many studies that reported on relation of pre‐ICH antiplatelet therapy with hematoma expansion had a significant proportion (30%–50%) of missing follow‐up imaging.^4^, ^6^, ^28^, ^29^, ^30^, ^31^ Therefore, the current study is a valuable addition to the literature on the effects of antiplatelet therapy in ICH. To the best of our knowledge, this study was also the only one to evaluate the effects of tranexamic acid on antiplatelet‐associated ICH.^32^

Because of the post hoc nature of the analyses, one limitation of the study was the findings are mainly hypothesis generating. We endeavored to reduce errors caused by multiplicity of testing by adhering to prespecified outcome measures and a statistical analysis plan. Another limitation was the type and number of antiplatelet agents, the duration of treatment, and the fact that the last dose was not recorded. Platelet activity may be suppressed more significantly in patients with dual or triple antiplatelet agents, while some newer antiplatelet agents, such as prasugrel or ticagrelor, are more potent than older ones, such as clopidogrel or aspirin.^33^ On the other hand, genetic polymorphism may lead to reduced efficacy of clopidogrel in some patients.^34^ The degree of platelet inhibition is therefore not known. In addition, intensive blood pressure lowering in the first 6 hours had been shown to reduce hematoma expansion.^35^ However, blood pressure levels and management within this early window was not recorded in our study.

## Conclusions

Pre‐ICH antiplatelet therapy is associated with an increased risk of hematoma expansion, neurological deterioration, death, and unfavorable functional outcome in spontaneous ICH. Trials of hemostatic therapies in ICH should include or even prioritize patients with antiplatelet therapy due to these reasons. Tranexamic acid did not have significantly greater benefit in patients with antiplatelet‐associated ICH, and its use in these patients needs to be further explored.

## Sources of Funding

This work was supported by the National Institute for Health Research Health Technology Assessment Programme (11_129_109) and the Swiss Heart Foundation.

## Disclosures

Dr Bath is Stroke Association Professor of Stroke Medicine and is a National Institute for Health Research senior investigator. He has received consulting fees from DiaMedica, Moleac, Nestle, Phagenesis, and Sanofi; he is an unpaid advisor to Platelet Solutions. Dr Desborough has received consultancy fees from Takeda and Portola. Dr Robinson is a National Institute for Health Research senior investigator. Dr Collins has accepted speaker's honoraria from Bayer, Boehringer Ingelheim, Pfizer, Daichii Sankyo, and Menarini. Dr Werring has received honoraria from Bayer, Alnylam and Portola. The remaining authors have no disclosures to report.

## Supporting information


Figures S1–S2
