# Supplementary material for: Outcomes in Antiplatelet‐Associated Intracerebral Hemorrhage in the TICH‐2 Randomized Controlled Trial
Source: J Am Heart Assoc. 2021 Feb 15;10(5):e019130. doi: 10.1161/JAHA.120.019130 (PMC8174262; doi:10.1161/JAHA.120.019130)

# SUPPLEMENTAL MATERIAL

Figure S1. Flow chart of patients included in analyses.

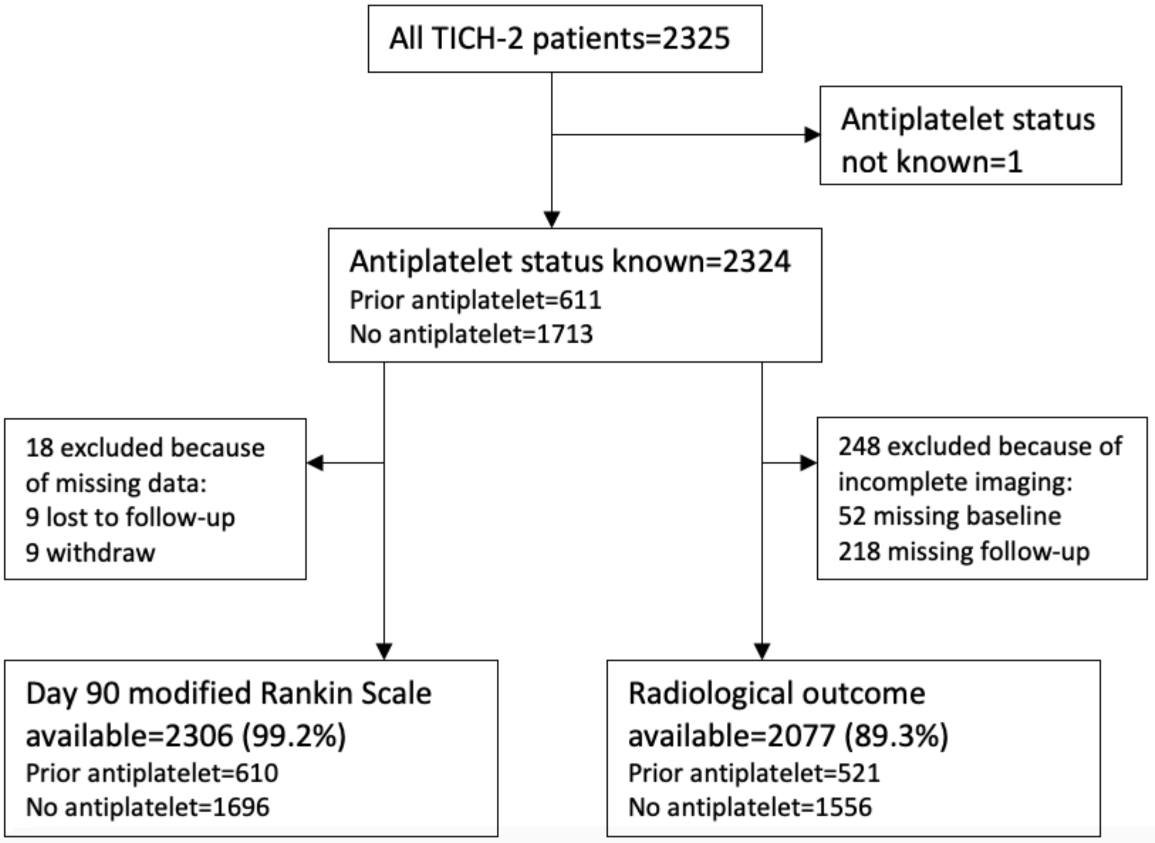

Figure S2. Ordinal regression analysis: adjusted 0.92, 95%CI 0.68-1.25; p=0.59
*Adjustment for age, sex, systolic blood pressure, NIHSS, onset to randomization time, intraventricular hemorrhage and country.

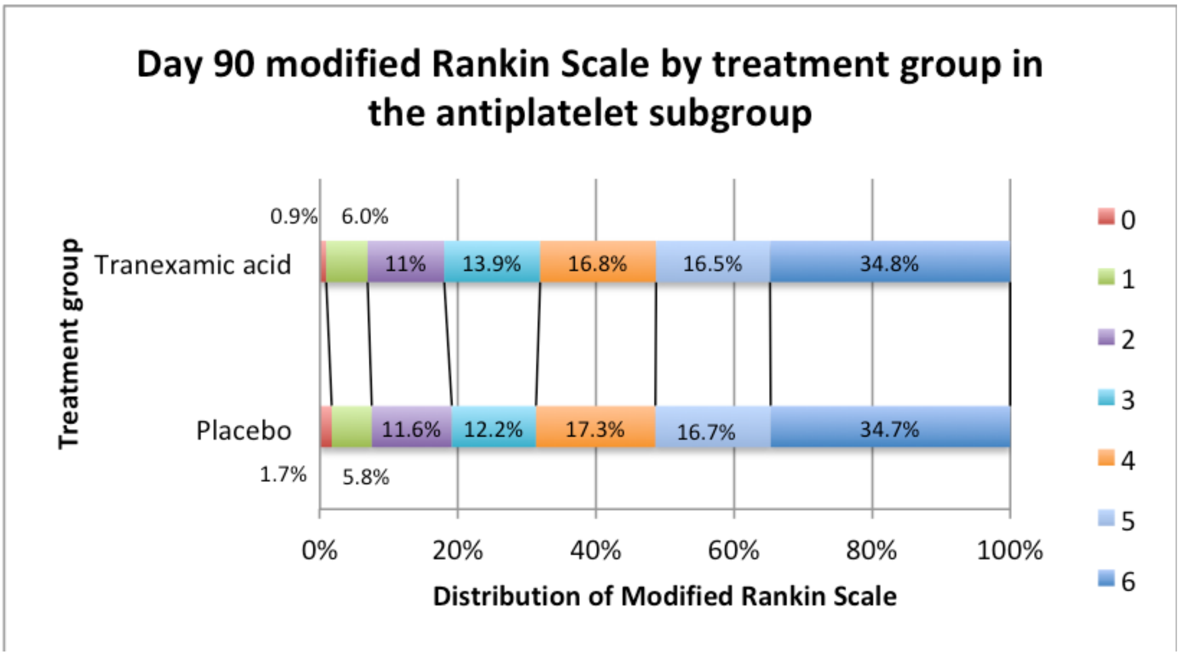

Supplement: Supplementary file 1 — Figures S1–S2 [file JAH3-10-e019130-s001.pdf]
